# Macrophage and fibro-adipogenic progenitor communication in skeletal muscle regeneration: tissue homeostasis and pathogenic remodeling

**DOI:** 10.1093/jleuko/qiag054

**Published:** 2026-04-28

**Authors:** Seily Shrestha, Abbey L Politeski, Sarah A Dick

**Affiliations:** Department of Biomedical and Molecular Sciences, Queen's University, 18 Stuart Street, Kingston, Ontario K7L 3N6, Canada; Department of Medicine, Queen's University, 18 Stuart Street, Kingston, Ontario K7L 3N6, Canada; Department of Biomedical and Molecular Sciences, Queen's University, 18 Stuart Street, Kingston, Ontario K7L 3N6, Canada

**Keywords:** fibro-adipogenic progenitors, macrophages, regeneration, skeletal muscle

## Abstract

Skeletal muscle regeneration depends on coordinated interactions between macrophages, fibro-adipogenic progenitors, and muscle stem cells. Following injury, macrophages transition from proinflammatory to anti-inflammatory phenotypes, regulating debris clearance, cytokine secretion, and the activity of fibro-adipogenic progenitors and muscle stem cells. Fibro-adipogenic progenitors transiently support muscle stem cell-mediated regeneration but, if not cleared appropriately, differentiate into fibroblasts or adipocytes, contributing to fibrosis and fatty infiltration. Dysregulated macrophage–fibro-adipogenic progenitor crosstalk drives pathological conditions, including Duchenne muscular dystrophy and age-related sarcopenia, where imbalances in cytokines and growth factors exacerbate maladaptive remodeling. Fibro-adipogenic progenitor-derived colony-stimulating factor 1 sustains macrophage survival while macrophage-derived signals, including tumor necrosis factor alpha and transforming growth factor beta, regulate fibro-adipogenic progenitor apoptosis, proliferation, and differentiation, shaping the regenerative niche. Single-cell and spatial transcriptomic approaches have revealed extensive heterogeneity among resident and infiltrating macrophages and fibro-adipogenic progenitor subsets, uncovering the molecular circuits underlying intercellular communication. Therapeutic strategies targeting cytokines and growth factors show promise in restoring balanced macrophage–fibro-adipogenic progenitor signaling, enhancing regeneration, and limiting fibrosis and fatty infiltration. Understanding the temporal dynamics of macrophage–fibro-adipogenic progenitor interactions is essential for developing interventions that preserve muscle homeostasis and counteract degenerative disease.

## Teaser

Skeletal muscle regeneration relies on dynamic macrophage–FAP crosstalk that coordinates the balance between regeneration and fibrosis, disruption of which drives maladaptive remodeling, highlighting therapeutic opportunities to restore the balance.

## Key concepts

- Macrophage phenotype determines FAP fate: proinflammatory macrophages induce FAP apoptosis, while anti-inflammatory macrophages promote FAP survival and differentiation.
- Dynamic macrophage–FAP crosstalk is essential for coordinated muscle regeneration and limiting fibrosis or fatty infiltration.
- Dysregulation of this reciprocal interaction contributes to pathological remodeling in aging and disease, such as sarcopenia and DMD.

## Open questions

- How do macrophages and FAPs interact in the skeletal muscle microenvironment?
- How do the timing and dynamics of macrophage–FAP interactions determine regenerative versus fibrotic outcomes?
- Can targeted modulation of macrophage–FAP crosstalk improve muscle repair without promoting fibrosis or fatty infiltration?

## 1. Introduction

Skeletal muscle is one of the most abundant tissues in the human body and is critical for locomotion, force generation, energy metabolism, and thermoregulation.^1,2^ Structurally, skeletal muscle is composed of multinucleated myofibers organized into fascicles and surrounded by layers of extracellular matrix (ECM) that provide structural support and facilitate communication between resident cell populations.^3^ In response to injury, skeletal muscle exhibits a remarkable capacity for regeneration through a tightly coordinated process involving inflammatory responses, activation of muscle stem cells (MuSCs), and interactions among immune and stromal cells within the tissue microenvironment.^4–8^ Following injury, immune cells rapidly infiltrate the damaged tissue, contributing to debris clearance, inflammatory signaling, and coordination of regenerative processes.^9^ Among these, macrophages are key regulators of muscle repair, controlling debris clearance, cytokine release, and interactions with MuSCs and stromal populations, including fibro-adipogenic progenitors (FAPs).^10–13^ Concurrently, FAPs expand and provide structural and paracrine support to promote MuSC activation and differentiation.^13^ Macrophages and FAPs engage in reciprocal interactions that shape each other's function and fate, collectively supporting a proregenerative environment. However, the specific signaling pathways that mediate this relationship, and how their interaction may influence the outcome of muscle regeneration, remain an active area of investigation.

Despite their central role, macrophage biology in skeletal muscle is highly complex and cannot be fully captured by traditional classification systems. Macrophages have been traditionally classified as M1 (proinflammatory) and M2 (anti-inflammatory).^14^ While this terminology provides a useful conceptual framework, it oversimplifies the complex and heterogeneous macrophage phenotypes that exist in vivo, where mixed activation states are common.^14^ A major limitation of early studies was the lack of tools available to distinguish infiltrating monocyte-derived macrophage (MDM) subsets from skeletal muscle resident macrophages (SMRMs), which were often overlooked. Single-cell RNA-sequencing (scRNA-seq) has revealed extensive heterogeneity among both SMRM and MDM subsets in uninjured and injured muscle (Table 1), highlighting the need to move beyond binary M1/M2 classifications to fully capture the immune complexity underlying regeneration.^15,21^

**Table 1:** Key macrophages subsets, their gene expression signatures, and proposed functions in skeletal muscle repair.

	Macrophage subset	Key markers	Functional roles in muscle	References
SMRM	TLF^+^	*Timd4* ^+^, *Lyve1*^+^, *Ccr2*^−^	Debris clearance	^15–17^
	MHC-II^hi^	*Timd4* ^−^, *H2-Ab1* (MHC-II)^+^, *Ccr2*^−^	Unknown	^15^
	CCR2^+^	*Timd4* ^−^, *Lyve1*^−^, *Ccr2*^+^	Unknown	^15^
MDM	Proinflammatory	*Ly6c* ^+^, *Chil3*^+^, *Spp1* (OPN)^+^	Promote inflammation, FAP activation	^18,19^
	Anti-inflammatory	*Il7r* ^+^, *Atf3*^+^	Debris clearance, resolution of inflammatory signaling	^20^
	Proregenerative	*Gpnmb* ^+^, *Mrc1* (CD206)^+^	myoblast fusion/efferocytosis, growth factor expression	^18,21^
	Disease associated	*Lgals3* (Gal-3)^+^, *Spp1* (OPN)^+^	Profibrotic/FAP activation and collagen deposition	^22,23^

Protein name indicated in brackets when different from the gene name.

While this cellular complexity is essential for effective regeneration following acute injury, it becomes disrupted in chronic conditions such as Duchenne muscular dystrophy (DMD) and age-related sarcopenia.^24,25^ DMD is a severe X-linked muscle-wasting disease caused by mutations in the dystrophin gene, leading to progressive muscle degeneration and loss of ambulation during adolescence.^26,27^ The *mdx* mouse serves as the primary preclinical model used to study disease mechanisms and therapeutic strategies in DMD.^28^ Aging similarly alters the regenerative environment, disrupting coordinated interactions among MuSCs, FAPs, and immune cells. In aged muscle, immune profiles shift, regenerative capacity declines, and MuSC proliferation is reduced, accompanied by chronic activation of inflammatory and stress pathways.^29^

Recent advances have begun to reveal the complexity of immune–stromal interactions during muscle regeneration.^30^ In this review, we focus on how macrophages influence tissue repair and fibrosis through their interactions with FAPs and discuss emerging tools for studying these processes. We highlight the interplay between macrophages and FAPs, examining their contributions to muscle regeneration and pathology, with emphasis on acute injury, DMD, and age-related sarcopenia. As anti-inflammatory and antifibrotic therapies are under active investigation for various muscle-related conditions, understanding these interactions may provide insights into novel strategies for enhancing muscle repair while limiting fibrosis.

## 2. The cellular niche in skeletal muscle homeostasis

**Figure 1:**
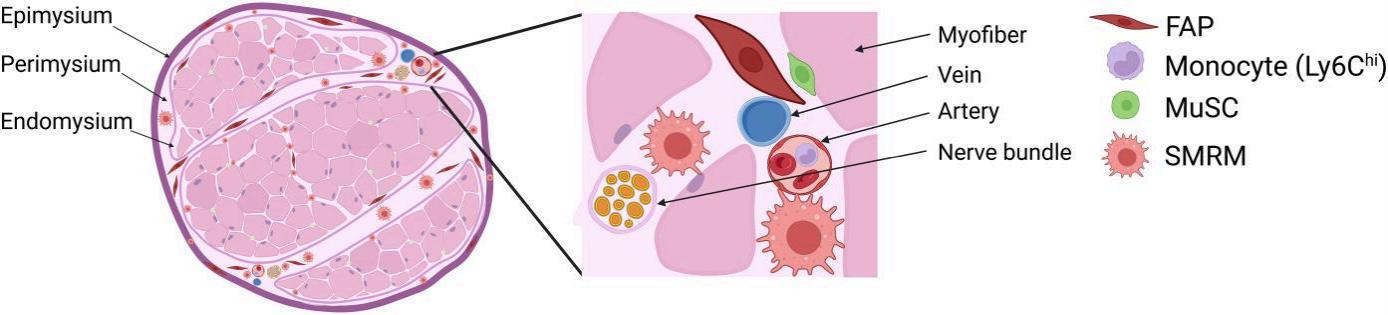
Spatial organization of skeletal muscle during homeostasis. In steady-state skeletal muscle, SMRMs and FAPs occupy overlapping interstitial areas across the epimysium, perimysium, and endomysium. SMRMs are present throughout the muscle bundles and perivascular space, while quiescent MuSCs lie adjacent to the plasma membrane of muscle fibers.

In healthy skeletal muscle, resident macrophages are distributed throughout the interstitial compartments, including the epimysium, perimysium, and endomysium,^31^ and have also been reported to exhibit perivascular localization.^32,33^ MuSCs reside between the basal lamina and the sarcolemma of myofibers, where they remain in a quiescent state until activated by injury or stress.^34^ The FAPs, a major stromal population in skeletal muscle, are primarily localized around blood vessels and within the interstitial space.^35–39^ Functionally, FAPs contribute to muscle development and regeneration by producing ECM components that support MuSC activation and myogenesis.^40,41^ Importantly, SMRMs and FAPs occupy overlapping interstitial niches, placing them in close spatial proximity and enabling functional interactions that contribute to tissue homeostasis (Fig. 1).

### 2.1. Macrophage and FAP heterogeneity

Tissue-resident macrophages (TRMs) have been shown to form heterogeneous populations consistent across tissues and species with distinct developmental origins and functional properties.^31,42^ Combined scRNA-seq and flow cytometric analyses have identified 3 conserved TRM subsets defined by the expression of 4 key markers: T-cell immunoglobulin and mucin domain-containing protein 4 (TIMD4); lymphatic vessel endothelial hyaluronan receptor 1 (LYVE1); Folate receptor 2 (FOLR2); and C-C motif chemokine receptor 2 (CCR2).^15^ The TLF^+^ subset (TIMD4 ^+^ LYVE1 ^+^ FOLR2 ^+^ CCR2^−^) is primarily embryonic-derived and maintained independently of circulating monocytes, whereas the remaining populations express higher levels of major histocompatibility complex class II (MHC-II) and include an MHC-II^hi^ subset (TIMD4^−^LYVE1^−^CCR2^−^) and a CCR2^+^ subset (TIMD4^−^LYVE1^−^CCR2^+^), with the latter exhibiting extensive replenished by monocytes and limited self-renewal capacity.^15,31^ Spatially, TRMs occupy distinct microanatomical niches. For example, in the lung, heart, adipose tissue, and dermis, the MHC-II^hi^ TRMs are closely associated with nerve bundles and fibers, while TLF^+^ TRMs are enriched around CD31^+^ blood vessels.^32^

Similar patterns of heterogeneity are observed in skeletal muscle, where SMRMs can be broadly categorized into TLF^+^, MHC-II^hi^, and CCR2^+^ populations.^10,15,31^ These SMRMs are ontogenetically and functionally distinct from MDMs recruited following injury.^31^ While MDMs have been extensively studied in the context of muscle regeneration, the roles of SMRMs remain comparatively less understood. Emerging evidence suggests that they contribute to muscle homeostasis by clearing apoptotic cells and maintaining tissue integrity.^16,17^ However, the precise spatial localization of all 3 SMRM subsets within skeletal muscle has yet to be fully characterized.

A similar degree of heterogeneity has also been described among fibroblast populations with distinct spatial and functional properties.^43^ In steady-state skeletal muscle, FAPs represent approximately 30% of stromal cells and are characterized by expression of platelet-derived growth factor receptor alpha (PDGFRα), stem cell antigen-1 (SCA-1), and cluster of differentiation 34 (CD34), along with stromal-associated genes including decorin (*Dcn*) and gelsolin (*Gsn*).^36,44^ FAPs have been further subdivided into 2 populations in noninjured muscle: dipeptidyl peptidase IV (DPP4)^+^ and C-X-C motif chemokine ligand 14 (CXCL14)^+^.^18^ DPP4^+^ FAPs expressed peptidase inhibitor 16 (*Pi16*) and wingless-related integration site 2 (*Wnt2*) and resembled the reticular interstitial adipose progenitors described in adipose tissue.^18^ CXCL14^+^ FAPs are enriched for genes encoding secreted enzymes such as *Enpp2*, *Crispld2*, and *Hsd11b1*, suggesting a distinct secretory phenotype.^18^ Another study similarly identified 2 distinct FAP subsets in both mice and humans, dividing them into lumican (LUM)^+^ and fibrillin 1 (FBN1)^+^ populations with differential collagen expression.^45^ LUM^+^ FAPs are primarily associated with collagen IV and XV in the basal lamina or endomysium and align closely with a gene expression profile similar to CXCL14^+^ FAPs.^45^ On the other hand, FBN1^+^ FAPs are enriched for collagen XIV in the perimysium.^45^ While it remains unclear how the FAP subsets identified in these 2 studies relate to one another, they are reminiscent of a conserved fibroblast signature identified across several tissues, which parsed resident fibroblast cells into a Pi16^+^ adventitial/vasculature subset and a collagen XV alpha1 (Col15a1)^+^ parenchymal fibroblast population.^43^ Despite these advances, the spatial organization and overlap of these heterogeneous FAP and SMRM subsets have yet to be fully characterized. However, emerging evidence suggests that their spatial co-localization may establish a microenvironment conducive to localized signaling interactions that support tissue homeostasis.

### 2.2. CSF1-PDGF reciprocal signaling between macrophages and FAPs

Beyond the muscle, macrophage–fibroblast interactions have been well studied and shown to sustain through reciprocal trophic signaling. Macrophage survival, proliferation, and self-renewal are critically dependent on colony-stimulating factor 1 (CSF1) signaling via the CSF1 receptor (CSF1R),^46^ whereas fibroblast survival relies on platelet-derived growth factor (PDGF) signaling via its receptor PDGFRα.^47^ Fibroblasts are a primary source of CSF1, while macrophages express high levels of *Pdgfb*, establishing a bidirectional trophic circuit between these populations.^12,48^ This expression pattern has been validated in both in vitro and in vivo systems.^12^

This interdependence is further reinforced by regulatory mechanisms, including Yes-associated protein 1 (YAP1)-dependent control of CSF1 expression via a fibroblast-specific enhancer located approximately 30 kb upstream of the *Csf1* gene.^49^ Functional evidence from co-culture studies demonstrates that macrophages and fibroblasts converge toward a stable population ratio over time, regardless of initial seeding ratios.^12^ This equilibrium depends on direct cell-cell contact to facilitate efficient growth factor exchange, constraints on cell proliferation, and negative feedback regulation of CSF1 via receptor-mediated internalization.^12^ A tightly regulated system that ensures the mutual maintenance of both cell types. Although this reciprocal communication has been described in various tissues such as the liver and spleen,^12,50,51^ its functional relevance in skeletal muscle, particularly for regulating FAP survival and proliferation, is only beginning to be revealed.

**Figure 2:**
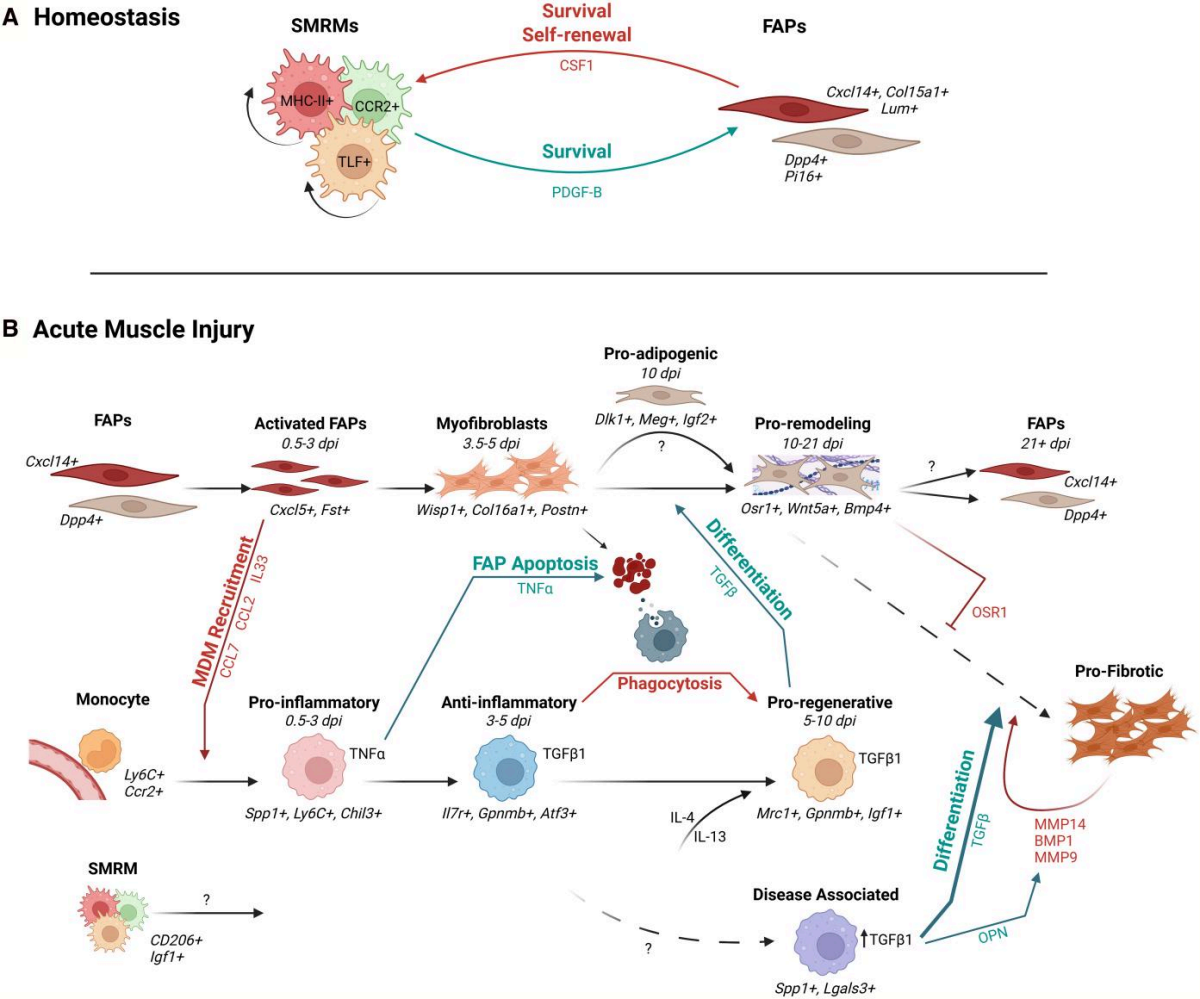
Cellular dynamics and macrophage–FAP signaling during homeostasis and regeneration following acute muscle injury. A) A SMRM-FAP circuit that establishes cell numbers in muscle is dependent on reciprocal expression of the growth factors CSF1 and PDGF-B. B) Following acute muscle injury, FAPs (*Cxcl14*^+^, *Dpp4*^+^) become activated (*Cxcl5*^+^, *Fst*^+^) and differentiate into myofibroblasts (*Wisp1*^+^, *Col16a1*^+^, *Postn*^+^). Activated FAPs promote monocyte and MDM recruitment through the secretion of CCL7, CCL2, and IL33. Recruited monocytes (*Ly6C*^+^, *Ccr2*^+^) differentiate into proinflammatory MDMs (*Spp1^+^*, *Ly6C^+^*, *Chil3*^+^), which secrete TNF-α to promote FAP apoptosis and restrict pathogenic FAP expansion. Subsequently, MDMs polarize toward an anti-inflammatory phenotype (*Il7r*^+^, *Gpnmb*^+^, *Atf3*^+^). IL-4 and IL-13 further promote macrophage polarization toward a proregenerative state (*Mrc1*^+^, *Gpnmb*^+^, *Igf1*^+^), which produces TGF-β1. TGF-β signaling can drive FAP differentiation toward a proremodeling (*Osr1*^+^, *Wnt5a*^+^, *Bmp4*^+^) phenotype. FAPs can also transiently adopt a proadipogenic (*Dlk1*^+^, *Meg*^+^, *Igf2*^+^) fate. In addition, disease-associated macrophages (*Spp1*^+^, *Lgal3*^+^) can arise, amplifying TGF-β signaling and reinforcing a profibrotic loop through MMP14, BMP1, and MMP9.

In support of this, Babaeijandaghi et al. demonstrated that in muscle, local CSF1 derived from PDGFRα^+^ FAPs is critical for the survival of all macrophage populations.^16^ However, this effect was only observed following CSF1 loss under long-term conditions, indicating that macrophage–FAP interaction is primarily required for long-term tissue maintenance. Among the various FAP subsets, DPP4^+^ FAPs were found to be spatially associated with proliferating (5-ethynyl-2′-deoxyuridine [EdU^+^]) resident TLF^+^ macrophages,^16^ implying that these cells may form localized niches that support macrophage self-renewal. These observations support that, analogous to other tissues, macrophages and FAPs in skeletal muscle engage in reciprocal trophic signaling, potentially involving PDGF-mediated support of FAPs by macrophages (Fig. 2A). The spatial organization of these populations further suggests that distinct FAP subsets establish specialized stromal niches that sustain macrophage maintenance, while macrophage-derived signals may in turn regulate FAP survival and function. Defining these reciprocal interactions will be critical for understanding how local microenvironments govern skeletal muscle regeneration and long-term tissue maintenance.

## 3. MDM–FAP crosstalk in muscle injury and degeneration

MDMs are key regulators of skeletal muscle regeneration, bridging early inflammation and tissue repair. Following injury, recruited MDMs undergo dynamic phenotypic transitions and engage in bidirectional crosstalk with FAPs, influencing their expansion and fate. In turn, FAPs modulate macrophage recruitment and polarization, forming a feedback loop that determines whether regeneration proceeds efficiently or shifts toward pathological remodeling. This section examines how MDM–FAP interactions are coordinated during acute injury and how their dysregulation contributes to disease and aging.

### 3.1. Cellular dynamics of skeletal muscle regeneration in acute injury

While SMRMs and FAPs maintain tissue homeostasis in uninjured muscle through localized CSF1–PDGF signaling, acute injury disrupts these steady-state niches and initiates a highly coordinated regenerative program involving both resident and recruited cell populations. Acute muscle injury is commonly modeled using chemical toxins such as cardiotoxin (CTX), notexin (NTX), and barium chloride (BaCl_2_), which induce synchronized muscle fiber damage and allow controlled investigation of regenerative responses.^52^ Injury activates quiescent MuSCs, leading them to proliferate and generate myoblasts, which then terminally differentiate and fuse to form functional myofibers,^53^ a process controlled by cytokines and growth factors from immune cells and FAPs, dependent on spatial and temporal interactions within the injured tissue (Fig. 2B).

Within 24 h following injury, neutrophils infiltrate the tissue, promoting inflammation and the recruitment of lymphocyte antigen 6 complex (Ly6C)^+^ monocytes. These monocytes differentiate into proinflammatory MDMs within 1 to 3 days post-injury, enriched in the genes chitinase 3-like 3 (*Chil3*) and secreted phosphoprotein 1 (*Spp1*), encoding for chitinase-like protein 3 (Ym1) and osteopontin (OPN), respectively.^18,19,54,55^ These secrete cytokines such as tumor necrosis factor alpha (TNF-α) and interleukin (IL)-1β, thereby amplifying the inflammatory response and facilitating the removal of necrotic debris through phagocytosis.^56^ Over the next several days, macrophages undergo dynamic phenotypic transitions that are essential for successful repair.^56^ Between days 3 and 5 post-injury, an anti-inflammatory Il7r^+^ MDM subset expressing high levels of glycoprotein nonmetastatic melanoma protein B (GPNMB) emerges and contributes to muscle repair through efferocytosis.^18,21^ Additionally, Patsalos et al. revealed that around day 4 post-injury, the macrophage population diversifies into distinct proregenerative transitional states, including resolution-associated macrophages (*Atf3*, *Apoe*, *Mmp12*), growth-factor expressing macrophages (GFEMs; expressing *Gpnmb*, *Gdf15*, *Igf1*, *Trem2*), and antigen-presenting macrophages (*Cd74*, *H2-Aa*).^20,57^ These likely represent a continuum of macrophage states, with activating transcription factor 3 (ATF3) acting as a transcription factor for *Gpnmb* and potentially other GFEM-associated genes,^20^ and the persistence of macrophages with high expression of antigen presentation genes beyond day 10 post-injury.^18^ Using spatial transcriptomics, these subsets are spatially organized within regenerative inflammation zones, where GFEMs preferentially localize near regenerating embryonic myosin heavy chain (eMHC)^+^ fibers, suggesting exchange of repair signals.^20^ These findings highlight the increasing appreciation that specialized macrophage subsets perform distinct regenerative functions.

Concurrently, FAPs rapidly proliferate within the first few days, becoming activated and enriched in follistatin (*Fst*) while upregulating chemokines including *Cxcl5*, *Cxcl3*, *Ccl7*, and *Ccl2*, likely contributing to the recruitment of immune cells via the chemokine receptor CXCR2 and CCR2 expressed on monocytes.^18,39,58–60^ Activated FAPs co-localize with macrophages within regenerating tissue,^61^ and further promote macrophage recruitment through autocrine IL-33-IL1RL1/ST2 signaling.^62^ Disruption of FAP-derived IL-33 was shown to impair macrophage recruitment in vivo.^62^ This coincided with elevated ECM gene expression (*Col1a1*, *Col3a1*) and impaired regeneration,^62^ highlighting a role for recruited macrophages in limiting fibrosis. These findings support the emerging view that FAPs function not only as stromal progenitors but also as key organizers of the early inflammatory microenvironment. In parallel, FAPs transiently support MuSCs, promoting their proliferation and differentiation to facilitate regeneration,^63^ highlighting their multifaceted role in coordinating both immune and myogenic responses.

By days 3.5 to 5, FAPs transition into a myofibroblast-like state with increased expression of WNT1 inducible signaling pathway protein 1 (Wisp1) and ECM remodeling genes, including *Postn*, *Col8a1*, *Col12a1*, *Col16a1*, and *Col11a1*, reflecting a shift toward tissue repair and matrix deposition.^18^ The magnitude of this response is kept in check by FAP apoptosis and removal, in a macrophage-mediated process.^64^ As tissue repair progresses toward resolution, the phagocytic activity of early-recruited macrophages supports their transition into the proregenerative phenotype (*Ly6C*^lo^*Mrc1*^+^), which predominates by day 7 post-injury.^19,31,56^ This transition represents a critical checkpoint in regeneration, shifting the tissue environment from inflammatory clearance toward regenerative signaling. This switch is further reinforced by IL-4 and IL-13 secreted from eosinophils and Th2 cells.^9,65–67^ These macrophages secrete regenerative mediators such as transforming growth factor-β1 (TGF-β1), insulin-like growth factor (IGF)-1, and IL-10 while upregulating arginase-1 (Arg1) and activating an important cellular energy sensor AMP-activated protein kinase-α1 (AMPKα1),^56,68–71^ thereby creating an environment that promotes MuSC-mediated myogenesis.^72^

From days 7 to 21 post-injury, FAPs and macrophages progressively return to a state resembling that of uninjured muscle.^18,44^ During this resolution phase, proinflammatory macrophages are largely cleared, and the tissue is dominated by regenerating myofibers.^18^ A small transient population of delta-like noncanonical notch ligand 1 (Dlk1)^+^ FAPs expressing *Meg* and *Igf2* appears around day 10, with a transcriptomic signature resembling a proadipogenic fate.^18^ At later time points, a small population of macrophages and odd skipped-related 1 (Osr1)^+^ FAPs expressing bone morphogenetic protein 4 (*Bmp4*) and *Wnt5a* persists, likely supporting ongoing tissue remodeling.^18,44^ When this tightly regulated process is disrupted, FAPs may fail to resolve properly and instead adopt pathogenic adipogenic or profibrotic phenotypes. This leads to excessive ECM deposition, fibrosis, and fatty infiltration, ultimately impairing MuSC-mediated regeneration and promoting chronic muscle dysfunction.^58,73,74^ These dynamics highlight that the timely resolution and re-establishment of stromal–immune niches is a central determinant of successful muscle regeneration.

The role of SMRMs in this process remains poorly understood, and it is unclear whether they represent a distinct population or are largely integrated into regenerative macrophage subsets, such as *Mrc1* (CD206)^+^ and IGF1^+^ macrophages. Evidence has indicated a critical role for SMRMs in debris clearance, as their loss results in impaired regeneration.^16,17^ Furthermore, in a zebrafish model of muscle regeneration, resident macrophages physically interacted with MuSCs and secreted trophic factors, including nicotinamide phosphoribosyltransferase (NAMPT), which binds the CCR5 receptor to regulate repair.^75^ These suggest an important role for SMRMs in regeneration; however, their interaction with FAPs in this context remains limited.

### 3.2. Macrophage derived TNF-α/TGF-β regulates FAP dynamics in the regenerating muscle

A central mechanism governing macrophage–FAP crosstalk during regeneration is the temporal shift from TNF-α driven inflammation to TGF-β-mediated tissue remodeling. Seminal work by Lemos et al. demonstrated that early inflammatory macrophages express TNF-α, peaking around 3 days post-injury.^64^ TNF-α levels subsequently decline over the next 48 h, during which time macrophage-derived TGF-β1 expression increases.^64^ This transition corresponds to the phenotypic switch from proinflammatory to anti-inflammatory macrophages around days 3 to 4 post-injury, which persists until at least day 7.^19,31,56^

Early TNF-α expression is critical to induce FAP apoptosis, facilitating the removal of excess FAPs and preventing the overabundance of ECM that can lead to muscle stiffness and impaired regeneration.^64,76^ Consistent with this role, anti-TNF antibody treatment with MP6–XT3 in mice leads to prolonged FAP persistence and excessive collagen deposition.^64^ Continued FAP accumulation is similarly seen in *Ccr2*^−/−^ mice, as CCR2^+^ recruited macrophages were suggested to be the main cellular source of TNF-α.^64^ Further, depletion studies demonstrate that macrophage ablation using a CD11b-diphtheria toxin (DT) mouse model results in persistent vascular cell adhesion molecule 1 (Vcam1^+^) inflammatory FAP populations and increased fibrosis following NTX-induced injury, highlighting the importance of macrophage-mediated FAP apoptosis.^77^

Notably, not all FAPs are deleterious, and it remains unclear which subsets are specifically targeted for apoptosis versus those that survive. Late anti-inflammatory CD206^+^ macrophage-derived TGF-β1 promotes the survival of remaining FAPs and drives their differentiation into proremodeling myofibroblasts.^64,78^ This transition is critical for promoting matrix remodeling and re-establishing muscle function.^64^ However, the precise role of CD206^+^ macrophages in regeneration remains an area of ongoing debate. While their emergence coincides with resolution of inflammation and initiation of tissue repair, TGF-β signaling is also a well-established driver of fibrosis, raising the possibility that the same macrophage population may promote either regenerative remodeling or pathological matrix deposition depending on the timing and magnitude of TGF-β activity. Indeed, disruption of this tightly regulated temporal balance, particularly through excessive or prolonged TGF-β signaling,^79^ can lead to pathological outcomes. Stepien et al. demonstrated that deletion of macrophage-derived TGF-β1 reduces fibrosis by decreasing FAP proliferation and enhancing regeneration, as shown by increased eMHC expression following ischemic injury.^80^ Additionally, studies in mouse models have shown that depletion of CD206^+^ macrophages, or inhibition of their TGF-β1 expression, reduces fibrosis, in part by relieving suppression of FAP-derived Fst and follistatin-like 3 (Fstl3) to promote a more regenerative environment.^78^ Similarly, the accumulation of anti-inflammatory or profibrotic macrophages, as observed in disease and aging, promotes persistent FAP activation and fibrotic matrix deposition.^81,82^ Given its pleiotropic effects, TGF-β activity within the regenerative niche is likely influenced by multiple signals, many of which remain poorly characterized. Even within a macrophage-dominated environment, additional stromal factors can modulate TGF-β signaling in FAPs. For example, the MuSC-derived factor decorin was shown to modulate TGF-β1-dependent signaling activity in a chicken model by sequestering TGF-β from its receptors, potentially influencing whether TGF-β1 drives matrix remodeling or pathological fibrosis.^83^ These findings suggest that while CD206^+^ macrophages, and other anti-inflammatory macrophages, likely play a role in coordinating the transition from inflammatory clearance to tissue remodeling, prolonged or dysregulated TGF-β signaling from certain populations may instead drive fibrotic outcomes.

Collectively, these studies highlight macrophage polarization as a central regulatory switch controlling FAP fate during regeneration. Successful muscle repair requires a tightly coordinated sequence in which early inflammatory macrophages restrict FAP expansion by controlling their apoptosis, followed by anti-inflammatory macrophages that support controlled stromal remodeling of specific FAP subsets that persist to this stage. Disruption of this temporal balance, whether through chronic inflammation, excessive TGF-β signaling, or stromal senescence, promotes persistent macrophage–FAP signaling loops that ultimately drive fibrosis and regenerative failure.

## 4. Macrophage–FAP dysregulation in pathogenic remodeling

We are now beginning to resolve how macrophage–FAP interactions mechanistically determine whether regeneration proceeds efficiently or shifts toward pathological remodeling. For example, long-term ablation of PDGFRα^+^ FAPs reduces muscle mass, myofiber cross-sectional area (CSA), and grip strength,^36^ likely due in part to altered macrophage populations. Furthermore, in mouse models of hindlimb ischemia injury, CSF1R blockade impairs the differentiation of Ly6C^hi^ proinflammatory to a Ly6C^lo^ anti-inflammatory macrophage phenotype, highlighting a role for CSF1 beyond homeostatic maintenance.^84^ When this phenotype switch is absent, impaired regeneration occurs, with increased necrosis, smaller fiber size, and fat accumulation,^56,85^ highlighting that this axis likely represents an important stromal mechanism through which FAPs regulate the inflammatory trajectory of regeneration.

### 4.1. Duchane muscular dystrophy

**Figure 3:**
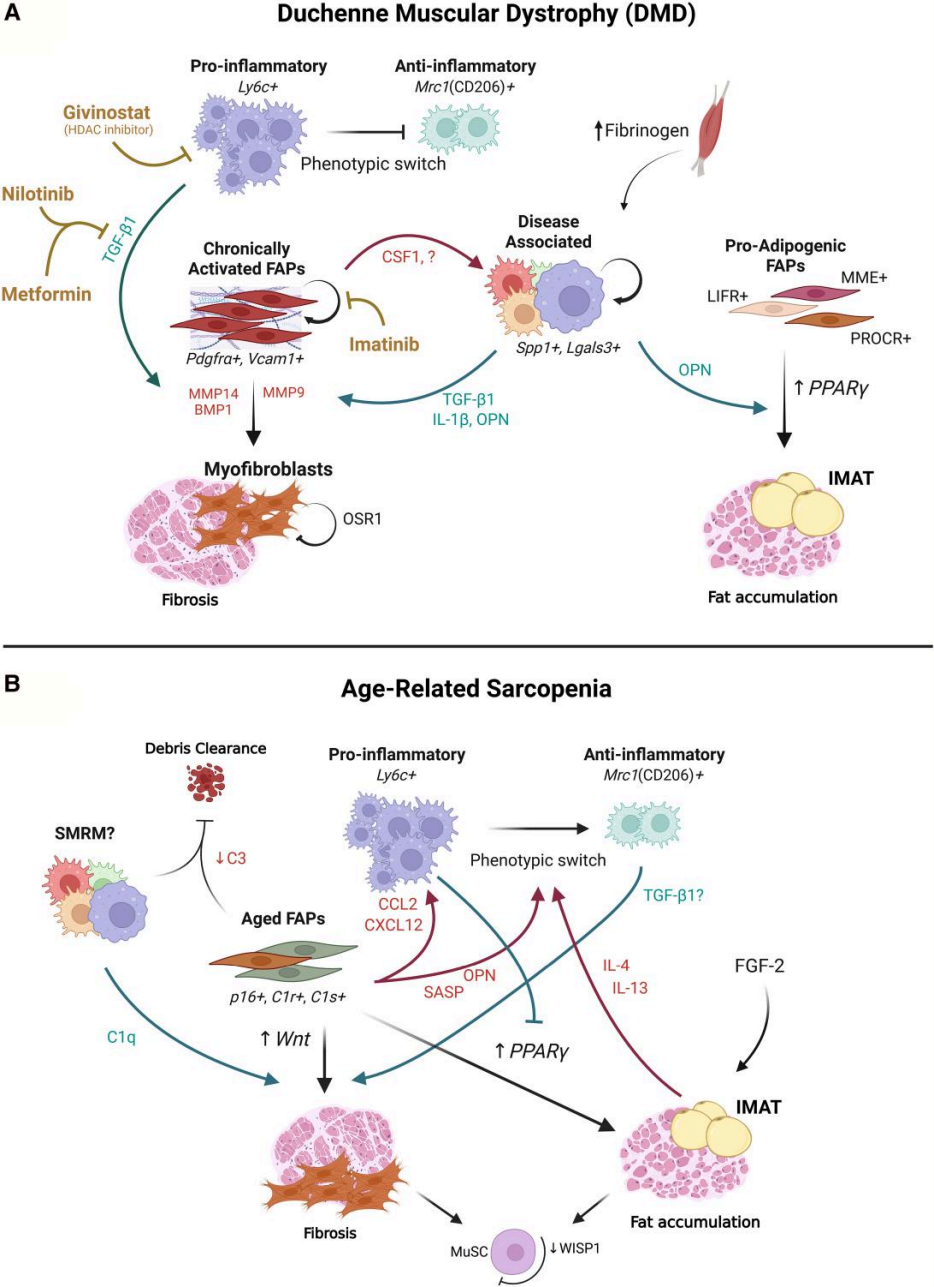
Dysregulation of macrophage–FAP communication in DMD and age-related sarcopenia. A) In dystrophic muscle, inflammatory MDMs (Ly6C^+^) secrete TGF-β1, which is activated by FAP-derived proteases including MMP9, MMP14, and BMP1. Enhanced TGF-β signaling promotes FAP differentiation into myofibroblasts, contributing to progressive fibrosis. The FAP transcription factor *Osr1* may bias FAPs toward a proremodeling state, partially limiting fibrosis. Fibrinogen accumulation further activates disease-associated macrophages (*Spp1^+^*, *Lgal3^+^*), inducing IL-1β and TGF-β1 production, amplifying TGF-β signaling. In parallel, *Spp1*^+^ macrophage-derived OPN promotes adipogenic differentiation of FAP populations. Elevated PPARγ expression in FAPs further drives the accumulation of IMAT. Targeted therapeutic strategies that modulate these pathways are shown. B) In aged skeletal muscle, there is excessive accumulation of anti-inflammatory macrophages (CD206^+^). Macrophages, suggested to be SMRMs, produce C1q, which activates canonical Wnt signaling in FAPs and promotes fibrotic responses. Aged FAPs exhibit elevated expression of the complement proteins C1r and C1s, with C1s being implicated in increased fibrosis. Reduced C3 levels associated with sarcopenia may impair regeneration through limiting macrophage-mediated phagocytosis of necrotic debris. Aged FAPs exhibit hallmarks of cellular senescence (*p16*) and an SASP. Together with OPN, these promote an anti-inflammatory macrophage polarization. Overall, FAP abundance declines with age, along with the secretion of key regenerative growth factors including WISP1, which is required to maintain MuSC stemness. Additionally, an age-associated increase in FGF-2 biases FAPs toward an adipogenic fate. IMAT accumulation further reinforces CD206^+^ macrophage polarization via IL-13, IL-4, and PPARγ signaling.

In DMD, persistent tissue damage leads to the dysregulation of the pro- to anti-inflammatory macrophage phenotype transition contributing to chronic inflammation, excessive fibrosis, and impaired regeneration, processes driven in part by disrupted macrophage–FAP communication^86^ (Fig. 3A). Unlike the transient inflammatory response following acute injury, early inflammatory interactions are amplified and sustained in dystrophic muscle. In the *mdx* mouse model, muscles exhibit persistent levels of both pro- and anti-inflammatory macrophages at 4 weeks of age.^87^

FAPs and immune cells represent the dominant populations in dystrophic muscles of (*mdx^5cv^*) mice.^88^ FAPs have been shown to promote the proliferation and pathogenic activation of resident Ly6C^lo^ macrophages via CSF1 signaling, in the absence of monocyte recruitment, as demonstrated in *mdx^5cv^/Ccr2^−/−^* mice.^89^ When macrophage recruitment is inhibited, elevated levels of CSF1, likely secreted by FAPs, drive the proliferation of SMRMs.^89^ These macrophages subsequently acquire a pathogenic disease-associated activation state, analogous to Ly6C^hi^ macrophages in wildtype muscle.^89^ Additionally, ablation of Ly6C^hi^ macrophage recruitment via CCR2 deficiency only transiently reduces muscle damage and fibrosis,^90^ suggesting compensatory mechanisms maintain macrophage–FAP signaling.

Notably, younger *mdx* mice display reduced regenerative capacity compared to adults, accompanied by increased inflammatory (iNOS^+^) macrophage infiltration and an elevated number of FAPs.^91^ Within degenerating regions, FAPs and macrophages are frequently found in close proximity,^92^ and the excessive FAP accumulation contributes to pronounced collagen deposition and fibrosis.^79,92,93^ These observations suggest that signaling pathways that normally coordinate early regeneration become dysregulated in dystrophic muscle,^86^ where sustained macrophage–FAP signaling may reinforce chronic inflammation and fibrotic remodeling rather than promoting resolution.

In support of this, *mdx* mice have also been shown to contain inflammatory Ly6C^+^ macrophages with elevated *ltbp4* expression, promoting the secretion of latent TGF-β1.^94^ This is subsequently activated by FAP-derived factors, specifically matrix metalloproteinase 14 (MMP14) and BMP1,^94^ amplifying fibrotic signaling in dystrophic muscle, which will be discussed in more detail below. The resulting active TGF-β1 stimulates collagen I production and promotes the differentiation of FAPs into alpha-smooth muscle actin (α-SMA)^+^ myofibroblasts in vitro.^94^ Through this mechanism, reciprocal signaling between macrophages and FAPs may reinforce fibrotic remodeling in dystrophic muscle, creating a microenvironment that perpetuates FAP activation and fibrosis.^91,95^

### 4.2. Age-related sarcopenia

As muscles age, their immunological profiles shift, resulting in a decline in regenerative capacity and reduced MuSC proliferation,^29^ with chronic activation of inflammatory and stress pathways.^96^ Concurrently, the number of FAPs declines with age, as well as the secretion of key growth factors, including vascular endothelial growth factor (VEGF) and Wnt,^96^ as well as WISP1, a factor critical for maintaining MuSC stemness.^97^ This reduction, combined with the accumulation of senescent-like MuSCs and progenitors, suggests a stalled self-renewal capacity.^98^ Furthermore, aged FAPs in human muscle have increased expression of the chemoattractant cytokine CCL2 and CXCL12, suggesting enhanced macrophage recruitment and inflammatory signaling.^96,99^ Interestingly, complement signaling represents one of the key modes of communication between FAPs and macrophages that becomes dysregulated with age. In skeletal muscle, macrophages, likely SMRMs, are the primary source of the complement component q (C1q), while aged FAPs exhibit elevated expression of the complement proteins C1r and C1s.^100^ During regeneration, C1q was shown to activate canonical Wnt signaling in FAPs, promoting fibrotic responses.^100^ FAP-specific deletion of C1s reduces fibrosis following BaCl_2_-injured aged muscle, further implicating complement signaling as a driver of age-associated fibrotic remodeling.^100^ Additionally, during the early inflammatory phase of injury in human skeletal muscle, FAP-derived complement factor 3 (C3) enhances macrophage-mediated clearance of necrotic myofibers by acting as an opsonization signal recognized through complement receptor 4 (CR4) subunits Integrin alpha X (ITGAX/CD11c) and Integrin beta 2 (ITGB2/CD18).^61^ Reduced circulating C3 levels associated with sarcopenia,^101^ raise the possibility that diminished complement-mediated crosstalk compromises efficient debris clearance and delays regenerative progression in aging muscle. Collectively, these findings suggest that aging disrupts coordinated macrophage–FAP communication that normally integrates debris clearance with ECM remodeling during muscle regeneration (Fig. 3B).

Aging can similarly promote conditions favoring excessive TGF-β activity. In mice, older muscles have been shown to produce elevated levels of TGF-β1 following CTX-induced injury.^102^ Additionally, aged FAPs exhibit hallmarks of senescence,^103,104^ including increased *p16* expression, telomere-associated foci (TAF), and γH2A.X-positive nuclei indicative of DNA damage in mice.^104^ ScRNA-seq data from aged mice as well as co-culture experiments demonstrate that senescent FAP exhibit a general senescence-associated secretory phenotype (SASP) that promotes anti-inflammatory macrophage polarization, characterized by increased CD206 expression.^99^ These observations raise the possibility that senescent FAPs actively reshape the macrophage niche in aged muscle. As CD206^+^ macrophages are known to produce profibrotic mediators such as TGF-β1, this suggests the existence of a reinforcing feedback loop in which senescent FAPs drive macrophage polarization that further promotes FAP activation and fibrotic remodeling. Such stromal–immune feedback mechanisms may therefore contribute to the progressive loss of regenerative capacity observed in aging skeletal muscles.

## 5. Regulation of FAP fate: adipogenesis vs. fibrogenesis

It is clear that FAPs play a central role in coordinating skeletal muscle regeneration through tightly regulated fate decisions, supporting activation and subsequent clearance.^63,64,76^ This transient response is essential for restoring tissue homeostasis as FAP persistence can lead to their adoption of alternative differentiation fates, primarily adipogenic or fibrogenic.^105^ These fate decisions are not cell-intrinsic alone but are heavily influenced by the surrounding immune microenvironment. As mentioned, this process is bidirectional. Altered immune signaling drives FAP fate, while FAP differentiation in turn reshapes macrophage phenotype, establishing a feedback loop that can either support regeneration or promote pathological remodeling.

### 5.1. Fibrotic fate

As discussed, in dystrophic muscle, sustained inflammatory signaling skews FAP fate toward fibrogenic differentiation, leading to excessive ECM deposition and impaired skeletal muscle regeneration.^22,106^ Specialized disease-associated macrophage subsets amplify pathological FAP activation in DMD. Among these, galectin-3^+^ (*Lgals3*^+^) macrophages are enriched in dystrophic muscle and colocalize with PDGFRα^+^ FAPs.^22^ These macrophages secrete OPN,^22^ which is found elevated in the serum and muscle biopsies of *mdx* mice and individuals with DMD following disease onset.^23^ OPN signals through CD44 and integrins on FAPs and was shown to enhance TGF-β activation in an MMP9-dependent manner.^22,106^ Sustained OPN signaling in DMD promotes chronic FAP activation and excessive collagen deposition.^22,106^ In support of this, experimental ablation of OPN reduces TGF-β activation, fibrosis, and muscle weakness.^23,107^ These findings highlight OPN as a key mediator linking macrophage inflammatory activity to FAP-driven fibrotic remodeling.

Although OPN depletion decreases the ratio of pro- to anti-inflammatory macrophages, these changes in TGF-β signaling appear independent of macrophage polarization,^107^ and additional extracellular signals further reinforce this profibrotic niche. For example, fibrinogen accumulation in DMD muscles activates macrophages through the Mac-1 receptor, inducing IL-1β and TGF-β1 production while simultaneously stimulating fibroblasts through αvβ3 integrin signaling.^108^ These interactions promoted an anti-inflammatory (CD206 ^+^ Arg1^+^) macrophage phenotype,^108^ further sustaining FAP activation and matrix deposition. This supports a model in which macrophage-derived inflammatory mediators act as upstream regulators of FAP fibrogenic activity in dystrophic muscle.

Consistent with these observations, dystrophic muscle contains an expanded population of Vcam1^+^ FAPs, a subset associated with enhanced fibrotic activity.^77^ However, FAPs are increasingly recognized as a heterogeneous population with distinct functional states, and it remains unclear which subsets are most responsive to OPN and other profibrotic signals. This heterogeneity suggests that macrophage-derived cues may selectively influence specific FAP populations, potentially driving expansion of subsets with greater fibrogenic potential. Determining the specific FAP populations that drive fibrosis versus those that retain regenerative potential could help target pathological remodeling while preserving beneficial stromal functions.

Beyond extracellular cues, intrinsic transcriptional programs also determine FAP fate. Loss of the transcription factor *Osr1* in FAPs skews them toward a profibrotic state, reinforcing pathological remodeling in injured muscle.^109^  *Osr1*-deficient FAPs exhibit increased TGF-β signaling, which promotes fibrosis-related gene expression, persistent ECM deposition, and impaired MuSC differentiation into myotubes.^109^ Co-culture experiments confirm reduced myogenic differentiation with *Osr1*-deficient FAPs, and pharmacological inhibition of TGF-β signaling partially rescues these defects.^109^ These findings highlight *Osr1* as an intrinsic regulator that limits FAP fibrogenic potential and supports a proregenerative niche, suggesting that both external macrophage-derived cues and cell-intrinsic transcriptional programs work together to determine whether FAPs support regeneration or fibrosis.

Aging further exacerbates fibrotic remodeling by altering both FAP populations and immune composition (Fig. 3B). Transcriptomic analyses indicate that aged muscle macrophages undergo substantial phenotypic remodeling, characterized by downregulation of resident markers such as *Lyve1* and *Folr2*, alongside increased expression of MDM-associated genes such as *Gpnmb* and *Spp1*, as well as proinflammatory mediators including *S100a8*, *S100a9*, and *Il1β*.^110^ Consistent with these changes, scRNA-seq of aged murine skeletal muscle reveals that aging leads to a loss of regeneration-associated regulatory macrophages expressing infiltrating MDM genes (*Ccr2*, *Ccr5*, *Cd14*, *Fn1*, *Thbs1*, *F10*), regulatory genes (*Arg1*, *Il10*, *Pf4*, *Vegfa*, *Spp1*, *Pdpn*, *Trem2*, *Hbegf*), and repair-associated macrophage genes (*Igf1*, *Vegfa*, *Pdgfa*, *Fabp5*, *Fabp4*, *Lpl*, *Ctsd*, *Ctsl*, *Ctsb*) that collectively lose phagocytic capacity with age, accompanied by expansion of infiltrating MDMs.^111^

Broader immune remodeling also occurs in aged muscle. Studies report reduced recruitment of proinflammatory macrophages but expansion of lymphoid-lineage cells,^111^ and anti-inflammatory macrophages in aged muscle,^98,112,113^ in addition to alterations in FAP composition and signaling. In aged human muscle, membrane metallo-endopeptidase (MME)^+^ FAPs, which possess strong adipogenic potential, are reduced, while remaining FAP populations exhibit enhanced profibrotic (TGF-β) and proinflammatory (IL-6) signaling pathways, with enhanced ECM remodeling.^96^ These coordinated changes in immune and stromal cell populations in aging promote a microenvironment that favors persistent fibrotic remodeling and impaired muscle regeneration.

Although aging and disease alter macrophage and FAP composition and signaling, how these cells communicate remains poorly understood. Identifying which macrophage-derived signals regulate FAP persistence or differentiation, and how FAPs reciprocally shape macrophage phenotypes, will be critical for understanding how disrupted immune–stromal crosstalk contributes to fibrosis and for identifying therapeutic targets to restore a regenerative niche in aged skeletal muscle.

### 5.2. Adipogenic fate

Beyond fibrosis, pathological conditions such as aging or muscular disease can also drive immune–stromal interactions that cause FAPs to adopt a fibro-fatty phenotype.^97,103,114,115^ This adipogenic shift contributes to the accumulation of intermuscular adipose tissue (IMAT)^116^ between muscle fibers,^117^ which negatively impacts muscle contractile function.^115^ The accumulation may result from reduced physical activity and muscle atrophy,^118–120^ leading to functional decline and an increased risk of physical dependency.^121^

This increased IMAT accumulation can be characterized by elevated expression of peroxisome proliferator-activated receptor γ (*PPARγ*) and CCAAT/enhancer-binding protein alpha (*C/EBPα*).^122^ Consistently, a FAP-specific PPARy knockout model reduced IMAT accumulation by blocking FAP differentiation into adipocytes without promoting a fibrogenic fate, resulting in increased CSA, myofiber density, and contractile force.^123^ Transplantation experiments further demonstrate that FAPs promote white adipose tissue formation when introduced into glycerol-injured muscle, but not into healthy muscle, emphasizing the importance of environmental signals in determining FAP fate.^39^ In humans, MME^+^ FAPs represent the main adipogenic subpopulation, giving rise to adipocytes during fatty infiltration.^124^ Their strong adipogenic potential is linked to reduced activity of the WNT signaling pathway, which normally suppresses adipogenesis, resulting in lower β-catenin levels and a greater tendency to differentiate into adipocytes.^124^

Several signaling pathways are involved in increased IMAT accumulation in aged muscles^116^ (Fig. 3B). For example, fibroblast growth factor-2 (FGF-2) promotes adipogenesis by upregulating miR-29a, which suppresses the adipogenic inhibitor secreted protein acidic and rich in cysteine (SPARC) and biases FAPs toward differentiation into adipocytes.^125^ These adipogenic FAP populations, in turn, can shape the immune composition within muscle. In mice, IMAT promotes CD206^+^ macrophage polarization via IL-13 and IL-4 signaling, suggesting the existence of a feedforward loop between adipogenic FAPs and macrophage polarization.^126^ Notably, in aged human muscle, CD206^+^ macrophages accumulate primarily within IMAT-rich regions of the perimysium but remain largely absent from MuSC niches, indicating a distinct spatial segregation of immune and regenerative compartments.^126^

Macrophage polarization remains a key regulator of FAP adipogenic fate. In vitro studies have shown that IL-1β-polarized macrophages suppress FAP adipogenesis and induce a proinflammatory gene profile (*IL-6*, *CXCL8*, *TNFA*) through activation of the anaplastic lymphoma kinase (ALK)/mothers against decapentaplegic homolog 2 (Smad2) pathway, highlighting direct macrophage–FAP crosstalk.^92^ Conversely, PPARγ expression in FAPs feeds back to influence macrophage polarization, supporting the transition from pro- to anti-inflammatory states. This has been shown in in vivo studies where PPARγ knockout mice exhibited impaired muscle regeneration alongside a disrupted macrophage phenotype switch.^127^

Interestingly, macrophage-derived OPN was also directly shown to contribute to adipogenic remodeling in skeletal muscle. Mouse studies show that *Spp1*^+^ macrophage-derived OPN promotes adipogenic differentiation of PDGFRα^+^ FAPs, which includes 2 subsets: leukemia inhibitory factor receptor (LIFR^+^) and protein C receptor (PROCR^+^) populations.^128^ Depletion of *Spp1*^+^ macrophages reduces IMAT accumulation in *mdx* muscle,^128^ linking macrophage-derived signals to both fibrotic and fatty infiltration in dystrophic muscle. Furthermore, anti-inflammatory macrophages have been shown to promote brown/beige fat differentiation of FAPs following tendon tear injury.^129^ Transplantation of anti-inflammatory macrophage exosomes was found to promote this effect, reducing muscle atrophy and fatty infiltration,^129^ representing a potential therapeutic avenue.

Adipogenic differentiation is also regulated by intrinsic signaling mechanisms within FAPs via the primary cilia. Following injury, the proportion of ciliated FAPs surges from 10% to 50%, and genetic ablation of FAP cilia reduces adipocyte formation by over 70% at both 7 and 21 days post-glycerol-induced injury.^130^ Similar effects were observed in the *mdx* mouse model, where the loss of FAP cilia reduced the number of FAPs and increased myofiber size.^130^ Mechanistically, this effect is driven through de-repression of the ciliary Hedgehog target TIMP3, which inhibits the proadipogenic enzyme MMP14 to block fat formation while increasing the prevalence of newly formed myofibers.^130^ Building on the established role of macrophage-derived factors in driving adipogenic differentiation of FAPs, it is plausible that these immune-mediated cues interface with ciliary signaling pathways to regulate FAP fate. This suggests that primary cilia may function as integrative hubs, coordinating both intrinsic and extrinsic (immune-derived) signals to precisely modulate adipogenic differentiation during muscle regeneration and in pathological contexts. Overall, dysregulation of macrophage–FAP interactions during aging and disease promotes pathological fatty infiltration, underscoring the importance of balanced immune–stromal signaling for maintaining skeletal muscle regeneration.

## 6. Therapeutic targeting of FAP–macrophage crosstalk

Most of the effort in therapeutically targeting macrophages and FAPs in muscle repair is in the context of muscle degeneration diseases, such as DMD. Although the majority of clinical trials on DMD focus on gene therapy, anti-inflammatory mediators and growth hormones are continuously being explored for their role in limiting muscle degeneration (Table 2). Among these, corticosteroids, such as prednisone, deflazacort, and vamoraline, represent the most common treatments prescribed and show improvements in muscle strength and independent movement in DMD patients.^131^ However, due to the long-term side effects of these treatments, identifying novel anti-inflammatory therapies that do not involve steroids is a current area of investigation.

**Table 2:** Summary of therapeutic treatments used to treat patients with DMD targeting fibro/adipogenic progenitor–macrophage crosstalk.

Treatment	Species	Mechanism	Effect	References
Corticosteroids (prednisone, deflazacort, vamoraline)	Human	Inhibition of NF-ΚB to reduce T cell proliferation, B cells and antibody production	Limit muscle inflammation and fibrosis; delayed onset of dilated cardiomyopathy; steroid-related negative effects, such as weight gain and behavioral changes	^131^
Givinostat	Human	HDAC enzyme inhibitor	Limit muscle inflammation and fibrosis	^132^
Imatinib	Mouse	P38 kinase inhibitor	Reduces hindlimb fibrosis and FAP proliferation	^133^
Metformin	Mouse	AMPK activator	Decreased macrophage-derived TGF-β1, reduced necrosis and fibrosis, improved muscle force and CSA	^94^
Nilotinib	Mouse	P38 kinase inhibitor	Increased FAP apoptosis	^64^

Beyond corticosteroids, tyrosine kinase inhibitors have been actively investigated for their role in limiting fibrosis via FAP-mediated pathways. Imatinib, a tyrosine kinase inhibitor that blocks PDGFRα signaling, reduces hindlimb fibrosis and FAP proliferation without impairing myoblast proliferation in *mdx* mice.^133^ Similarly, nilotinib, another tyrosine kinase inhibitor, has been tested as an antifibrotic therapy in *mdx* mice, where its inhibition of TGF-β signaling through p38 kinase in Ly6C^hi^ macrophages increased TNF-mediated FAP apoptosis and reduced collagen I deposition.^64^ Furthermore, specific inhibitors of the CSF1R, a tyrosine kinase receptor for CSF1 signaling, are currently in clinical testing^134^ to modulate macrophage reprogramming in dystrophic tissues, where they have demonstrated a protective effect against eccentric contraction-induced muscle injury.^17^

Despite enormous progress, there remains a need for therapies that move beyond broad immunosuppression toward more targeted strategies that preserve regenerative capacity while limiting fibrosis. One promising approach is metformin, an AMPK activator widely used as an antidiabetic drug.^94^ Preclinical testing in *mdx* mice demonstrated that metformin was able to decrease macrophage-derived TGF-β1, reduce necrosis and fibrosis, and improve muscle force and CSA, while reducing the number of MuSCs.^94^ This was accompanied by a shift in macrophage polarization, with an increased proportion of CD206^+^ macrophages and a reduced proportion of TNF-α^+^ macrophages.^94^ Recently, the histone deacetylase (HDAC) enzyme inhibitor Givinostat was approved for treatment in DMD patients over 6 years old.^135^ It was found to effectively limit muscle inflammation and fibrosis, slowing DMD progression.^132^ Together, these studies highlight the therapeutic potential of targeting macrophage-driven pathways to modulate the regenerative microenvironment.

As we increase our understanding of the complex interplay between macrophage and FAP crosstalk in coordinating muscle regeneration and fibrosis, novel targets of this signaling network represent a promising future strategy to preserve muscle function. Direct modulation of FAP fate through PPARγ or TGF-β pathway inhibitors in human cells can further restrict adipogenic or fibrogenic differentiation, consistent with the observation that IL-1β and IL-4 polarized macrophages influence FAP outcomes in both acute injury and dystrophic contexts.^92^ Manipulating these cytokine pathways to balance FAP fate may therefore be a viable approach to limiting fibrotic accumulation. In support of this approach, Lemos et al. further showed that inhibition of TGF-β1, both in vitro and in vivo in *mdx* mice, increases FAP apoptosis^64^ leading to improved regeneration. As *Spp1*^+^ macrophages promote both adipogenic and fibrogenic FAP phenotypes through different signaling pathways,^22,99,106,110,128^ targeting SPP1 or its receptors could mitigate both fibrotic and adipogenic remodeling. Overall, these studies demonstrate that pharmacological modulation of macrophage–FAP crosstalk, whether through targeting the TGF-β pathway, SPP1, PDGFRα, and CSF1 pathways, holds strong translational promise for mitigating fibrosis and adipogenic remodeling, restoring macrophage balance, and enhancing muscle regeneration.

### 6.1. Exercise induced benefits

In addition to pharmacological interventions, exercise remains one of the top therapies to promote muscle health.^136^ Evidence suggests this is at least in part via its therapeutic effect on muscle repair through modulation of macrophage and FAP dynamics. A recent meta-analysis of over 7,000 human samples revealed that exercise induces a transient proinflammatory macrophage response within 6 h post-exercise.^137^ This phenomenon resolves within 24 h, followed by activation of anti-inflammatory phenotype.^137^ Notably, individuals who engage in long-term training exhibit an enrichment of proreparative, anti-inflammatory macrophages following the proinflammatory polarization, suggesting that repeated exercise promotes a proregenerative macrophage profile.^137,138^

Exercise also influences FAP behavior during muscle repair. In mice, a 14-day exercise regimen increased senescence markers in FAPs following acute injury, supporting their efficient clearance.^138^ This was accompanied by increased numbers of regenerating fibers, fiber CSA, and enhanced muscle strength.^138^ However, these benefits were lost in the setting of chronic inflammatory myopathy (CIM), where FAPs are resistant to TNF-induced apoptosis,^138^ highlighting the importance of macrophage-mediated FAP clearance. Pharmacological activation of AMPK using 5-aminoimidazole-4-carboxamide-1-β-d-ribofuranoside (AICAR) to promote FAP senescence in CIM restored muscle regeneration to levels comparable to baseline controls, effectively mimicking the regenerative effects of exercise.^138^ Although exercise alone was insufficient to exhibit beneficial effects in the CIM model, the improvement in regeneration observed in the acute injury setting highlights the importance of FAP clearance in maintaining a proregenerative niche and warrants further investigation in other disease models and aging.

Beyond its effects on acute regeneration, long-term exercise also prevents adverse tissue remodeling, including fibrosis and fatty infiltration, in mouse models of muscle atrophy and acute injury.^139^ This protective effect is mediated by the myokine musclin (encoded by *Ostn*), which is upregulated following exercise and is primarily produced by myofibers.^139^ Musclin inhibits FAP proliferation and promotes their apoptosis through Filamin A Interacting Protein 1 Like (FILIP1L) upregulation, and the Musclin/FILIP1L pathway further enhances macrophage phagocytosis of apoptotic FAPs via CD47 downregulation.^139^ These findings identify exercise-induced myokine signaling as an upstream regulator of macrophage-mediated stromal remodeling.

Together, these findings position exercise as a potential key regulator of the macrophage–FAP reparative niche. Acute exercise transiently activates proinflammatory macrophages, followed by a shift toward a proreparative, anti-inflammatory phenotype. The distinct macrophage polarization induced by exercise is amplified in individuals who engage in regular training, but this effect becomes less pronounced with aging.^137^ These observations underscore the importance of sustained physical activity in maintaining a healthy immune–stromal environment. Promoting consistent exercise in the aging population may therefore help preserve healthy macrophage–FAP functions, prevent pathological remodeling, and promote efficient muscle regeneration throughout life. While current evidence supports the independent roles of macrophages and FAPs in exercise-induced regeneration, direct investigation of exercise-mediated macrophage–FAP communication remains limited. Future studies to explore this crosstalk in greater detail will help clarify how exercise-mediated signaling between these cell types contributes to muscle regeneration.

## 7. Concluding remarks

Skeletal muscle homeostasis and regeneration depend on tightly coordinated communication between macrophages, FAPs, and MuSCs. Proinflammatory macrophages limit excessive fibroblast expansion through TNF-α-mediated FAP apoptosis, whereas anti-inflammatory macrophages promote FAP survival and matrix production via TGF-β1 signaling, together maintaining a balance between effective regeneration and fibrosis.^64^ Dysregulation of this network leads to impaired repair, increased fibrosis, and fat infiltration, with outcomes further influenced by factors such as aging and disease.

Despite recent advances, key questions remain regarding how microenvironmental cues shape macrophage polarization and FAP behavior during muscle repair. It remains unclear which upstream signals promote macrophage polarization toward profibrotic phenotypes, and whether macrophages associated with chronic muscle diseases such as DMD represent distinct populations or maladaptive extensions of anti-inflammatory, proreparative macrophages. Progress in this area is hindered by the lack of consensus on macrophage subset nomenclature, with overlapping transcriptional and functional definitions (eg, “M2-like,” “anti-inflammatory,” or “alternatively activated”), making it difficult to delineate which macrophage populations mediate specific effects on FAPs. Collectively, current evidence establishes macrophage–FAP crosstalk as a central regulatory axis in skeletal muscle regeneration while highlighting important gaps in our understanding of how macrophage heterogeneity and niche signals determine regenerative versus fibrotic outcomes.

Although key signaling pathways governing macrophage–FAP communication have been identified, the precise molecular mechanisms and temporal coordination of these interactions remain incompletely understood. In addition, MuSCs engage in bidirectional crosstalk with both FAPs and macrophages, forming a tightly integrated regenerative triad that shapes muscle repair outcomes. Future studies are required to map these interactions across distinct temporal phases of injury and repair, and to determine how their disruption contributes to chronic damage, aging-associated decline, and muscular dystrophies.

Overall, *Spp1*^+^ macrophages influence 2 distinct FAP phenotypes, fibrogenic and adipogenic, but the molecular mechanisms governing this dual regulation remain unclear. Current research suggests that persistent crosstalk between inflammatory macrophages and FAPs, rather than macrophage phenotype alone, maintains a pathological niche that favors fibrosis. These findings highlight how dysregulated macrophage–FAP communication lies at the core of DMD pathology, where sustained inflammatory and profibrotic signaling exacerbates fibrosis and fatty infiltration, ultimately compromising the regenerative capacity of dystrophic muscle.

Emerging single-cell and spatial profiling approaches now offer unprecedented opportunities for understanding macrophage heterogeneity and intercellular communication within regenerating muscle. Applying these technologies across time, aging, and disease contexts will be critical for refining current models of immune–stromal crosstalk and identifying therapeutic strategies to enhance regeneration while mitigating fibrosis.

## Data Availability

No new data were generated or analyzed in this study. Data sharing is not applicable to this article.

## Abbreviations

ATF3: Activating transcription factor 3
ALK: Anaplastic lymphoma kinase
AMPKα1: AMP-activated protein kinase-α1
BaCl2: Barium chloride
BMP: Bone morphogenetic protein
CCR2: C-C motif chemokine receptor 2
CD: Cluster of differentiation
Chil3: Chitinase 3-like 3
Col15a1: Collagen type XV alpha 1
CSF1: Colony-stimulating factor 1
CSF1R: Colony-stimulating factor 1 receptor
CSA: Cross-sectional area
CXCL: C-X-C motif chemokine ligand
C1q: Complement component q
C3: Complement factor 3
C/EBPα: CCAAT/enhancer-binding protein alpha
Dcn: Decorin
Dlk1: Delta like noncanonical notch ligand 1
Dpp4: Dipeptidyl peptidase 4
DMD: Duchenne muscular dystrophy
DT: Diphtheria toxin
ECM: Extracellular matrix
EDU: 5-Ethynyl-2′-deoxyuridine
eMHC: Embryonic myosin heavy chain
FAP: Fibro-adipogenic progenitor
FBN1: Fibrillin 1
FGF-2: Fibroblast growth factor 2
FILIP1L: Filamin A Interacting Protein 1 Like
FOLR2: Folate receptor 2
Fst: Follistatin
Fstl3: Follistatin-like 3
Gal-3: Galectin-3
GFEM: Growth factor expressing macrophages
GPNMB: Glycoprotein nonmetastatic melanoma protein B
Gsn: Gelsolin
HDAC: Histone deacetylase
IGF-1: Insulin-like growth factor 1
IL: Interleukin
IMAT: Intramuscular adipose tissue
ITGAX: Integrin alpha X
ITGB2: Integrin beta 2
LIFR: Leukemia inhibitory factor receptor
Ly6C: Lymphocyte antigen 6 complex
LUM: Lumican
LYVE1: Lymphatic vessel endothelial hyaluronan receptor 1
MHC-II: Major histocompatibility complex class II
MDM: Monocyte-derived macrophage
MME: Membrane metallo-endopeptidase
MMP: Matrix metalloproteinase
MuSC: Muscle stem cell (satellite cell)
NAMPT: Nicotinamide phosphoribosyltransferase
NTX: Notexin
OPN: Osteopontin
Osr1: Odd skipped-related 1
PDGF: Platelet-derived growth factor
PDGFRα: Platelet-derived growth factor receptor alpha
Pi16: Peptidase inhibitor 16
PPARγ: Peroxisome proliferator-activated receptor gamma
PROCR: Protein C receptor
SASP: Senescence-associated secretory phenotype
SCA-1: Stem cell antigen-1
SMAD2: Mothers against decapentaplegic homolog 2
SMRM: Skeletal muscle-resident macrophage
SPARC: Secreted protein acidic and rich in cysteine
Spp1: Secreted phosphoprotein 1
TAF: Telomere-associated foci
TIMD4: T-cell immunoglobulin and mucin domain-containing protein 4
TGF-β: Transforming growth factor beta
TNF-α: Tumor necrosis factor alpha
TRM: Tissue-resident macrophage
VEGF: Vascular endothelial growth factor
Vcam1: Vascular cell adhesion molecule 1
WISP1: WNT1-inducible signaling pathway protein 1
WNT: Wingless-related integration site
YAP1: Yes-associated protein 1
Ym1: Chitinase-like protein 3
α-SMA: Alpha-smooth muscle actin
